# Barrier to Autointegration Factor Becomes Dephosphorylated during HSV-1 Infection and Can Act as a Host Defense by Impairing Viral DNA Replication and Gene Expression

**DOI:** 10.1371/journal.pone.0100511

**Published:** 2014-06-19

**Authors:** Augusta Jamin, Prasanth Thunuguntla, April Wicklund, Clinton Jones, Matthew S. Wiebe

**Affiliations:** 1 School of Veterinary Medicine and Biomedical Sciences, University of Nebraska, Lincoln, Nebraska, United States of America; 2 Nebraska Center for Virology, University of Nebraska, Lincoln, Nebraska, United States of America; University of Alberta, Canada

## Abstract

BAF (Barrier to Autointegration Factor) is a highly conserved DNA binding protein that senses poxviral DNA in the cytoplasm and tightly binds to the viral genome to interfere with DNA replication and transcription. To counteract BAF, a poxviral-encoded protein kinase phosphorylates BAF, which renders BAF unable to bind DNA and allows efficient viral replication to occur. Herein, we examined how BAF phosphorylation is affected by herpes simplex virus type 1 (HSV-1) infection and tested the ability of BAF to interfere with HSV-1 productive infection. Interestingly, we found that BAF phosphorylation decreases markedly following HSV-1 infection. To determine whether dephosphorylated BAF impacts HSV-1 productive infection, we employed cell lines stably expressing a constitutively unphosphorylated form of BAF (BAF-MAAAQ) and cells overexpressing wild type (wt) BAF for comparison. Although HSV-1 production in cells overexpressing wtBAF was similar to that in cells expressing no additional BAF, viral growth was reduced approximately 80% in the presence of BAF-MAAAQ. Experiments were also performed to determine the mechanism of the antiviral activity of BAF with the following results. BAF-MAAAQ was localized to the nucleus, whereas wtBAF was dispersed throughout cells prior to infection. Following infection, wtBAF becomes dephosphorylated and relocalized to the nucleus. Additionally, BAF was associated with the HSV-1 genome during infection, with BAF-MAAAQ associated to a greater extent than wtBAF. Importantly, unphosphorylated BAF inhibited both viral DNA replication and gene expression. For example, expression of two regulatory proteins, ICP0 and VP16, were substantially reduced in cells expressing BAF-MAAAQ. However, other viral genes were not dramatically affected suggesting that expression of certain viral genes can be differentially regulated by unphosphorylated BAF. Collectively, these results suggest that BAF can act in a phosphorylation-regulated manner to impair HSV-1 transcription and/or DNA replication, which is similar to the antiviral activity of BAF during vaccinia infection.

## Introduction

Herpes simplex virus type 1 (HSV-1) is a common human pathogen; approximately 60% of the U.S. population is infected and even greater rates of infection are estimated worldwide [1]–[3]. Recurrent ocular HSV-1 infection is a leading cause of infectious corneal blindness in industrialized nations [4]–[6] and HSV-1 induced encephalitis (HSE) is a severe form of focal necrotizing encephalitis that affects at least 2,000 individuals each year in the U.S.[7]–[9]. HSV gene expression in productively infected cells is temporally regulated in three distinct phases: immediate early (IE), early (E), or late (L) [10]. IE transcription does not require protein synthesis and is stimulated by a viral tegument protein, VP16 [11]. E gene expression is dependent on at least one IE protein, and generally E genes encode nonstructural proteins that play a role in viral DNA synthesis. L gene expression is maximal after viral DNA replication, requires IE protein production, and L proteins comprise the virion particle.

Specific functions involving regulation of gene expression and interaction with host restriction factors have been ascribed to many viral proteins present early in the infection. For example, four IE genes encode ICP0, ICP4, ICP22, and ICP27. ICP4 [12]–[14] and ICP27 [15]–[17] are required for efficient virus growth in tissue culture. In general, ICP4 represses IE gene expression [13], [18]–[22] but activates E or L gene expression by interacting with RNA polymerase II transcription factors [19], [23]. ICP27 redistributes small nuclear ribonucleoprotein complexes, interferes with splicing of IE transcripts, and promotes E and L poly-A site selection [24], [25]. ICP0 can activate expression of all classes of viral genes, in part because it increases steady-state levels of mRNA [26]. ICP0 also binds several cellular proteins: 1) elongation factor 1δ [27], 2) cyclin D3 [28], 3) an ubiquitin-specific protease [29], [30], and 4) PML [31]–[33]. Interactions between ICP0 and chromatin-remodeling enzymes activate viral transcription [34]–[38]. Secondly, ICP0 alters a complex that inhibits gene expression (REST/CoREST/histone deacetylase repressor complex) [35]. In addition to these proteins, HSV-1 encodes several other factors that interfere with antiviral responses, thus promoting productive infection [39]–[41]. The complex interactions that occur between HSV-1 and host factors, including innate and intrinsic immune regulators, determine the outcome of an infection.

Barrier-to-autointegration factor (BAF/BANF1) is an essential, highly conserved metazoan protein with multiple functions linked to maintaining the integrity of the cellular genome. BAF can interact with double-stranded DNA in a sequence-independent manner and homodimerizes to crossbridge and condense DNA while forming higher order nucleoprotein complexes [42]–[45]. BAF also interacts with many cellular proteins including LAP2/emerin/MAN1 (LEM) domain proteins that reside in the nuclear envelope, histones, lamins, transcription factors, and DNA damage response (DDR) proteins [46]–[51]. Using these interactions, BAF is thought to act as a tethering protein to bring together chromatin DNA and LEM proteins during late stages of mitosis when the nuclear envelope (NE) is being reassembled. The importance of BAF during mitosis is underscored by evidence that misregulation of these BAF-dependent processes leads to chromosome segregation and NE defects, mislocalization of LEM proteins, and embryonic lethality in *Caenorhabditis elegans* and *Drosophila melanogaster* [52]–[55].

Interestingly, BAF is also a host defense effector against vaccinia virus in the cytoplasm. Specifically, BAF is capable of inhibiting vaccinia DNA replication [56] and transcription of vaccinia genes [57]. This host defense activity of BAF depends on its DNA binding and bridging properties and is blocked through phosphorylation by the vaccinia-encoded B1 protein kinase [58]. Phosphorylation of BAF by either the viral B1 or cellular protein kinase VRK1 (vaccinia related kinase 1) strongly inhibits the ability of BAF to bind DNA, thus regulating BAF-mediated assembly of nucleoprotein complexes in both the cytoplasm and nucleus [56], [59], . Phosphorylation of BAF by both viral and cellular kinases occurs at its N-terminus on Thr-2, Thr-3, and Ser-4 [60], [61]. While these sites are highly conserved throughout evolution and regulate BAF in multiple organisms [62]–[64], much remains to be learned about how they control the antiviral capabilities of BAF.

Although BAF has antiviral activity directed against vaccinia, it is not clear whether this activity is restricted to this poxvirus, perhaps because its replication cycle occurs in the cytoplasm, or whether it has antiviral activity directed against nuclear DNA viruses as well. Consequently, we performed studies to examine whether BAF has the potential to interfere with HSV-1 productive infection. We found that HSV-1 infection led to the dephosphorylation and rapid nuclear localization of BAF suggesting that it sensed nuclear viral DNA. Interestingly, overexpression of a BAF mutant that cannot be phosphorylated, the FLAG-BAF-MAAAQ mutant, inhibited HSV-1 replication in mouse L929 cells as compared to control cells or cells overexpressing epitope-tagged wild type BAF. Relative to other viral proteins examined, expression of VP16 and the ICP0 protein was strongly inhibited in cells expressing the FLAG-BAF-MAAAQ mutant. These experiments suggest that unphosphorylated BAF stably interacts with nuclear HSV-1 DNA and interferes with productive infection by impairing both viral DNA replication and gene expression. Finally, these studies also suggest that phosphorylation of wild type BAF down-regulates its antiviral function, thus allowing for efficient HSV-1 replication.

## Materials and Methods

### Cell Culture

Mouse fibroblast L929 cells (obtained from the American Type Culture Collection) were maintained in Dulbecco’s modified Eagle’s medium (DMEM) supplemented with 10% fetal bovine serum (FBS; Atlanta Biologicals) and penicillin-streptomycin and incubated at 37°C in a 5% CO_2_ atmosphere.

### Mutagenesis and Cloning of BAF Mutants

To construct the vector for 1xFLAG-tagged BAF and 1xFLAG-tagged BAF-MAAAQ, pcDNA5/FRT/TO/3XFLAG-BAF [56] and pcDNA5/FRT/TO/3XFLAG-MAAAQ [58] were used as a template for by PCR using outside primers Bam Kozak 1XFlag (5′-CTCGAGGGATCCGCCACCATGGATTACAAGGATGACGATGAC-3′) and BAF BamHI 3′ (5′-GCAGGATCCTCACAAGAAGGCGTCGCAC-3′. PCR products were BamHI-digested and ligated to BamHI-digested pHAGE-HYG-MCS (pHM) lentiviral vector (a generous gift from Dr. Paula Traktman, Medical College of Wisconsin) to generate pHM/HYG/1xFLAG-BAF and pHM/HYG/1xFLAG-MAAAQ.

### Production of Stably-expressing Cells

The stable overexpression of BAF in L929 cells was performed by using a lentivirus expressing 1xFlag-BAF or BAF mutant as previously described in Ibrahim et al., 2011. For transduction, L929 cells were seeded in 35 mm dishes at ∼1×10^6^ per well. The next day, medium was replaced with 1 mL of lentivirus supernatant for 24 h. Medium was then replaced with fresh media for an additional 24 h. Cells were then passaged in media containing 200 µg/ml of hygromycin to select for stable lentiviral integration and a polyclonal population of cells was employed for all experiments.

### Virus Infections and Viral Yield Assay

A HSV-1 recombinant virus LAT-GFP, strain McKrae, and Vaccinia (VACV) Cts2 virus were used for these studies. In cultured cells, LAT-GFP grows like wt McKrae. All infections were done at 37°C. For viral yield analysis, L929 cells (3×10^6^) were infected with either virus at MOI = 0.01 for 48 h. For HSV-1 infection, following cell harvest, cells and media were collected and frozen at −80°C. Samples were freeze-thawed three times followed by centrifugation to pellet cell debris. Supernatant was used for titration on Vero cells at 37°C. For vaccinia infection, following cell harvest, cells were pelleted and suspended in 10 mM Tris (pH 9). Samples were freeze-thawed three times prior to titration on BSC40 at 32°C. For DNA replication analysis, L929 cells (3×10^6^) were infected with MOI = 0.1 for 3 h and 24 h. Following cell harvest, cells were pelleted and DNA purified using the GeneJET Whole Blood Genomic DNA Purification Mini Kit (Thermo Scientific #K0782) prior to qPCR analysis. For immunofluorescence analysis, L929 cells were infected with MOI = 1 for 6 h. For western blot analysis, L929 cells were infected with MOI = 1 for the indicated times prior to harvest.

### Subcellular Fractionation

L929 cells (2×10^6^) were collected by scraping and washed in PBS. Subcellular fractionations were performed as described in (Wassler et al., 1987) with minor modification. Cell pellets were treated with cell lysis buffer (50 mM Tris-HCl pH 6.8, 2 mM MgCl_2_, 150 mM NaCl, 0.5% saponin, protease inhibitor, and phosphatase inhibitor) on ice for 10 min. Soluble fractions were collected following centrifugation at 2000 rpm at 4°C for 10 min. The insoluble pellets were further treated with Triton lysis buffer (50 mM Tris-HCl pH 6.8, 2 mM MgCl_2_, 75 mM NaCl, 0.2% Triton X-100, 0.1% SDS, protease inhibitors, and phosphatase inhibitors) on ice for 10 min. TX-100 soluble fractions were collected following centrifugation at 10,000 rpm at 4°C for 10 min. To each fraction, 2X protein sample buffer was added followed by loading onto a 18% SDS-PAGE gel and electrophoresis performed.

### Immunofluorescence

Cells were fixed in 4% paraformaldehyde in phosphate-buffered saline (PBS) (10 mM Na_2_HPO_4_.7H_2_O, 1 mM KH_2_PO_4_, 2 mM KCl, 140 mM NaCl, pH 7.4) at room temperature for 20 min. Following fixation, cells were permeabilized with 0.2% Triton X-100 in PBS for 10 min at room temperature. Cells were then incubated with M2 FLAG antibody at a dilution of 1∶300 for 1 h at room temperature. Following washing with PBS, cells were then incubated with Alexafluor 488-conjugated goat α-mouse-488 (Invitrogen) in PBS at 1∶400 for 1 h at room temperature. Fluorescence images were then taken by indirect fluorescence on an inverted (Olympus IX 81) confocal microscope.

### Immunoblotting Analysis

To examine protein expression in uninfected or infected cells, L929 cells (1×10^6^) were harvested, pelleted, and suspended in 300 µl of SDS sample buffer (100 mM Tris pH 6.8, 2% β-mercaptoethanol, 2% SDS, 32.5% glycerol, bromophenol blue) supplemented with 10 units of Benzonase as described previously [57]. Lysates were resolved on 18% SDS-PAGE gel when analyzing BAF and resolved on 10% SDS-PAGE gel when analyzing HSV-1 proteins. When examining protein expression following infection of transduced cells, cells were washed 2X with PBS and harvested in 300 µL RIPA buffer (50 mM Tris.HCl pH 8, 150 mM NaCl, 2 mM EDTA pH 8, 1% NP-40, 0.5% sodium deoxycholate, 0.1% SDS, and protease inhibitor). Cells were disrupted by sonication for 20 sec and incubated at an end-to-end rotator at 4°C for 30 min. Protein extracts were obtained following centrifugation at 13,000 rpm at 4°C for 10 min. Protein amount was quantified using Bradford assay and 50 µg lysate were resolved on 10% SDS-PAGE gel. All protein was transferred to PVDF membrane. Blots were then incubated with the appropriate antibodies prior to signal development with chemiluminescent reagents. Quantifications of the chemiluminescence signal were performed by using Bio-Rad ChemiDoc™ XRS instrument and Bio-Rad ImageLab™ software. The primary antibodies used are as follows: β-tubulin 1∶20,000 (Sigma #T7816), FLAG M2 monoclonal 1∶8000 (Sigma #F1804), BAF 1∶5000 (custom antibody [57]), phosphospecific BAF 1∶1500 (custom antibody [65]), ICP0 1∶100 (Santa Cruz Biotechnology #sc-53070), ICP4 1∶100 (Santa Cruz Biotechnology #sc-69809), ICP27 1∶100 (Santa Cruz Biotechnology #sc-17544), ICP8 1∶100 (Santa Cruz Biotechnology #sc-53329), TK 1∶100 (Santa Cruz Biotechnology #sc-28038), gD 1∶100 (Santa Cruz Biotechnology #sc-21719), and VP16 1∶100 (Santa Cruz Biotechnology #sc-17547).

### ChIP Assays

Chromatin immunoprecipitation was performed as described in Ibrahim et al., 2013 [57] with the following minor modifications. Cross-linking was performed with 0.75% PFA for 5 min. In addition, after overnight incubation of lysates with antibodies and a 2 h incubation with Dynabeads, beads were washed 3 times with final wash buffer (20 mM Tris-HCl pH 8.0, 500 mM NaCl, 2 mM EDTA pH 8, 1% Triton X-100, and 0.1% SDS) followed by elution of bound complexes. Immunoprecipitated and input material was analyzed by quantitative PCR (StepOne Plus Real Time PCR, Applied Biosystems).

### DNA/RNA Purification and qPCR

Viral DNA was extracted using GeneJET Whole Blood Genomic DNA Purification Mini Kit (Thermo Scientific #K0782) or QIAamp DNA Blood Mini Kit (Qiagen #51106). For ChIP analysis, DNA was purified by glass bead purification using Agarose Dissolving Buffer (Zymo Research #D4001-1-50).

qPCR was performed using SYBR®Green PCR Master Mix (Applied Biosystems #4309155 or Bio-Rad iTaq Universal SYBR®Green Supermix #172-5121). Serial dilutions were included in each qPCR run to develop a standard curve and determine the PCR efficiency of the primer sets in that experiment set. qPCR analyses were performed using 1 µL of purified DNA and 1 µM of each primers as followed: ICP0 R0 N1 (5′-AATGGGCAACCCCGGTATTC-3′) and

ICP0 R0 N2 (5′-GGAACCCCAGGGGAGTGGTTAC-3′),

Actβ-fwd-mouse (5′-GGTCATCACTATTGGCAACG-3′) and

Actβ-rev-mouse (5′-CGTCACACTTCATGATGGAATTG-3′),

qpcrNEO-UP (5′-CTTGCTCCTGCCGAGAAAGT-3′) and

qpcrNEO-DOWN (5′-TTCGCTTGGTGGTCGAATG-3′).

### Statistics

All experiments shown were performed in three or more independent assays. Error bars shown in bar graphs represent standard deviations from the mean. The p-values indicated were calculated using the Student’s t-test. For western blot analyses, a blot representative of at least three experiments is shown.

## Results

### HSV-1 Infection Induces Dephosphorylation of BAF

**Figure 1:**
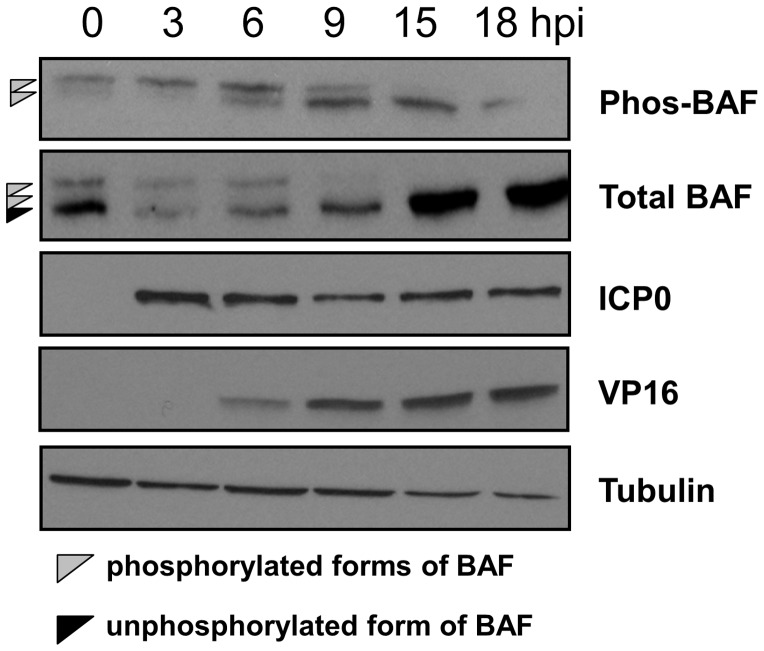
HSV-1 infection induces dephosphorylation of BAF. (A) Western blot analysis of cellular BAF and viral proteins upon infection of L929 cells. Cells were infected with HSV-1 virus at MOI = 1 for the indicated times prior to harvest. Following cell harvest, protein samples were resolved by SDS-PAGE and transfer to membrane. Membranes were incubated with antibodies specific to the proteins indicated at left. Data were obtained from three independent experiments and representative blots are shown.

In this study we tested the hypothesis that BAF can impair the lifecycle of a nuclear virus, HSV-1 for example, in a manner regulated by phosphorylation and/or localization of BAF. For this study, we have employed L929 cells because BAF possesses potent anti-poxviral activity in this cell type, strongly impairing both DNA replication and transcription of vaccinia genes when BAF is not phosphorylated [57]. Building on previous data that phosphorylation of BAF is altered during poxvirus infection, we examined the effects that HSV-1 infection has on BAF phosphorylation. For this study, antibodies specific to total BAF and phosphorylated BAF were employed. As described previously, BAF protein can be separated into two to three distinct protein bands of approximately 10 kDa in size [56], [59], [60]. Each distinct band detected by BAF-specific antibodies corresponds to specific BAF phosphorylation states in which the protein migration decreases as BAF becomes more phosphorylated. This property of BAF allows us to monitor BAF phosphorylation using the ‘total BAF’ antibody, which is able to recognize the fastest migrating unphosphorylated form of BAF as well as two shifted phosphorylated species. We also employ a phosphospecific BAF antibody, which recognizes only the two post-translationally modified forms of BAF due to the presence of one or more phosphate additions to BAF. Expression of the viral immediate early protein ICP0 and late protein VP16 were also monitored in this experiment as indicators of progress through the viral lifecycle. As shown in Figure 1, there is a marked reduction in BAF phosphorylation beginning at 6 hpi and continuing during all later time points. This is indicated by the loss of the slowest migrating band detected by the phosphospecific BAF antibody as well as the loss of the shifted, phosphorylated form of BAF (gray arrowheads) and complementary increase in unphosphorylated BAF (black arrowhead) detected by the total BAF antibody at 9 to 18 hpi. As expected ICP0 and VP16 protein expression were not readily detected in mock-infected cells. However, ICP0 was readily detected by 3 hours after infection and VP16 was not detected until 6 hours after infection. Similar levels of protein were loaded in each lane because tubulin protein expression was detected in each lane. These data demonstrate that HSV-1 infection triggers a decrease in steady state levels of BAF phosphorylation.

### Stable Expression of a Constitutively Unphosphorylated Form of BAF in L929 Cells

**Figure 2:**
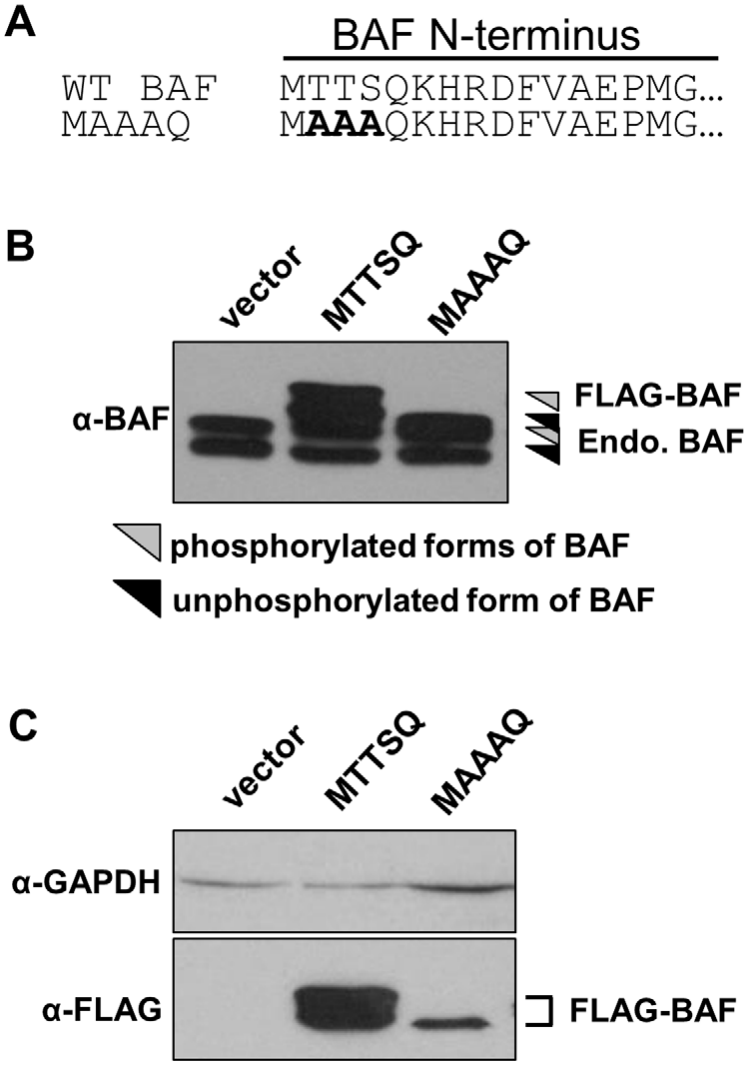
Stable expression of BAF proteins in L929 cells. (A) Point mutations were introduced to BAF N-terminal residues Thr2, Thr3, or Ser4 as indicated in the amino acid alignment. In each case the residue were mutated to A (alanine). (B–C) Representative western blot analysis of whole cell lysates from cells stably-expressing BAF-MTTSQ and BAF-MAAAQ. B) Anti-BAF antibody recognizes both endogenous BAF protein (arrowheads) and FLAG-tagged proteins (bracketed). C) Lysates from the same blot probed either withαGAPDH (top) or αFLAG (bottom).

To determine how dephosphorylated BAF may impact HSV-1, we stably expressed epitope tagged versions of wild type BAF or a BAF phosphorylation mutant in L929 cells. A FLAG epitope was chosen because FLAG-BAF has been utilized in multiple studies examining the phosphorylation of BAF, which have validated that it is regulated by viral and cellular enzymes similarly to untagged native BAF [56], [60], [61], [63]. We have also found that in multiple cell types, FLAG-BAF is localized to both the nucleus and cytoplasm, and can inhibit DNA replication and gene transcription of vaccinia virus in a manner dependent on the B1 kinase [56], thus indicating that FLAG-BAF can be regulated through phosphorylation as native BAF is. Specifically, to block BAF phosphorylation, we substituted the amino acids at known BAF phosphorylation sites (Thr2, Thr3, and Ser4) to an alanine (A) (Figure 2A), thus expressing a constitutively unphosphorylated form of BAF (FLAG-BAF-MAAAQ), and have cloned the BAF sequence encoding a wild-type N-terminus (MTTSQ) in the same vector for comparison. Both constructs were expressed using a lentiviral expression system, which allows for stable integration into the host genome and hygromycin resistance. Following antibiotic selection of transduced cells, expression of endogenous BAF and each FLAG-BAF protein was verified by immunoblot analysis with an anti-BAF antibody (Figure 2B). Consistent with these migration patterns, the epitope-tagged BAF-MTTSQ and BAF-MAAAQ also displayed distinct electrophophoretic mobilities reflecting the amount of negatively changed amino acids at their N-terminus regardless of whether the western blot was probed with the anti-BAF or anti-FLAG antibody (Figure 2B and C). These results confirm the stable expression of epitope tagged wild type and mutant BAF in our L929 cells, with FLAG-BAF-MAAAQ expressed at 20% of the level of FLAG-BAF-MTTSQ.

### Phosphorylation Modulates the Subcellular Localization of BAF in L929 Cells

**Figure 3:**
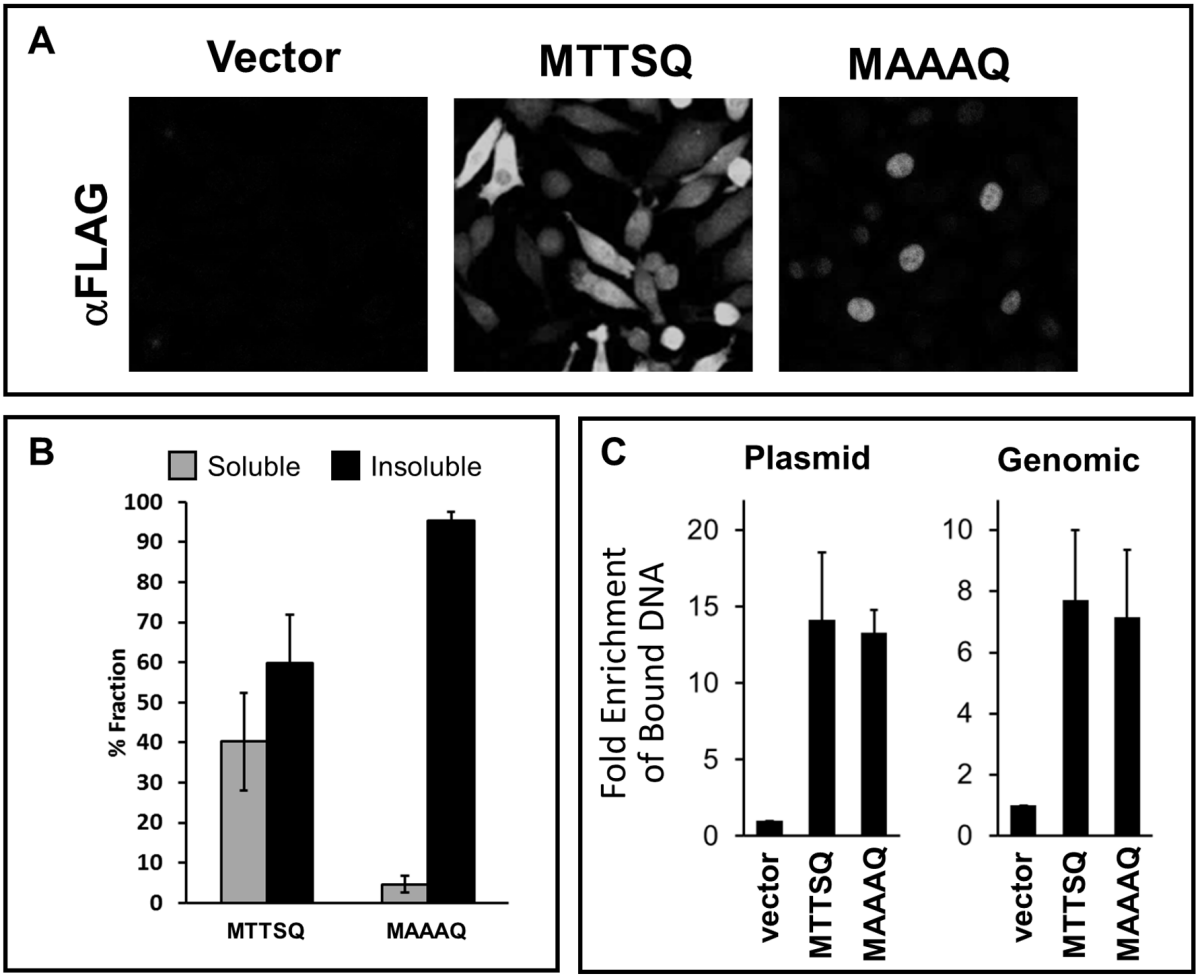
Subcellular distribution and DNA-binding activity of BAF mutants. (A) Immunofluorescence analyses of FLAG-BAF-MTTSQ and FLAG-BAF-MAAAQ with an anti-FLAG antibody (AlexaFluor488-conjugated secondary antibody). (B) Subcellular fractionation analyses of cells indicated in (A). Cell lysates were fractionated to saponin-soluble cytosolic fraction (grey bars) and insoluble nuclear fraction (black bars). Each fraction was analyzed by western blot with an anti-FLAG antibody and quantified with ImageLab software (BioRad) (n = 3). % fraction was calculated from the amount of protein on each fraction relative to the total protein level. Error bars represent standard deviations. (C) ChIP analyses of FLAG-BAF-MTTSQ and BAF-MAAAQ. Cells were transfected with 150 ng of pUC-Neo plasmid DNA for 24 h followed by fixation, immunoprecipitation with anti-FLAG antibody, and reverse crosslinking of protein-DNA complexes. Purified DNA was analyzed by qPCR using primers specific for the pUC-Neo plasmid (Plasmid) or the β-actin locus of chromatin DNA (Genomic). Fold enrichment was obtained relative to empty vector control and normalized to input DNA. Error bars represent standard deviations.

Previous studies as well as our own recent data [65] indicate that the subcellular localization and DNA binding activity of BAF can depend on its phosphorylation state [60]. Therefore, we compared the localization and DNA binding activity of FLAG-BAF-MTTSQ and FLAG-BAF-MAAAQ in uninfected cells. We began by analyzing the subcellular distribution of our stably expressed proteins by an immunofluorescence assay using an anti-FLAG antibody (Figure 3A). Like the native endogenous BAF [55], [58], [66]–[68], FLAG-BAF-MTTSQ is expressed in the nucleus, nuclear envelope and cytoplasm, and such localization is also consistent with the published results on GFP-tagged and 3xFLAG-tagged BAF [56], [60], [69], [70]. With regard to the mutant BAF, we found FLAG-BAF-MAAAQ primarily expressed in the nucleus. Next, to complement our immunofluorescence analyses of these proteins, we performed subcellular fractionation assays followed by immunoblot analyses. First, cells were lysed with the pore-forming agent saponin, which preferentially disrupts the plasma membrane, followed by treatment of the saponin-insoluble fraction with Triton X-100 and 0.1% SDS to complete disruption of the nuclear membrane [71]. As expected, FLAG-BAF-MTTSQ was distributed in both the saponin soluble (40%) and insoluble (60%) fractions, consistent with its presence in both the cytoplasm and nucleus (Figure 3B). In contrast, more than 95% of FLAG-BAF-MAAAQ was present in the TX-100/SDS soluble nuclear fraction, consistent with the immunofluorescence data above finding that FLAG-BAF-MAAAQ is largely nuclear. Thus, these results confirm and expand the evidence that BAF phosphorylation affect its subcellular distribution.

Based on previous evidence that BAF phosphorylation resulted in reduced affinity for DNA *in vitro* [60], we hypothesized that FLAG-BAF-MTTSQ may exhibit reduced DNA binding activity in cells as compared to FLAG-BAF-MAAAQ. To test this, we examined the DNA-binding activity of both proteins to either genomic DNA or foreign DNA (plasmid) via chromatin immunoprecipitation (ChIP) analysis. In this assay, L929 cells expressing empty vector control, FLAG-BAF-MTTSQ or FLAG-BAF-MAAAQ were transfected with 150 ng pUC-Neo plasmid for 24 h, and subsequently fixed with paraformaldehyde. Our recent study demonstrated that transfected DNA co-precipitates with FLAG-BAF-MTTSQ in ChIP analysis [57], validating plasmid as a source of foreign DNA that can be bound by BAF. After harvest and lysis of fixed cells, lysates were subjected to immunoprecipitation of FLAG-tagged BAF followed by reverse-crosslinking of protein-DNA complex and qPCR analysis. qPCR was performed on purified DNA by using primers specific for either the Neo^R^ gene or the β-actin locus, which was chosen to represent a region of cellular chromatin. Fold enrichment was calculated relative to our empty transduction vector control. From these analyses, we found that FLAG-BAF-MTTSQ interacted with plasmid DNA with an enrichment of ∼14-fold (Figure 3C, left). FLAG-BAF-MAAAQ interacts with plasmid DNA with a 13-fold enrichment, similar to FLAG-BAF-MTTSQ. With respect to binding to chromatin DNA, we found a similar trend of DNA binding between the BAF proteins. Specifically, both FLAG-BAF-MTTSQ and FLAG-BAF-MAAAQ were able to immunoprecipitate the β-actin locus with similar efficiency (Figure 3C, right). In sum, these data show that in uninfected cells both FLAG-BAF-MTTSQ and FLAG-BAF-MAAAQ immunoprecipitated similar amounts of transfected or genomic DNA despite their different subcellular localization. However, as we have not normalized for the lower expression of FLAG-BAF-MAAAQ in these cells, we may be underestimating the binding efficiency of FLAG-BAF-MAAAQ using this approach.

### HSV-1 Virus Production is Inhibited by Unphosphorylatable BAF

**Figure 4:**
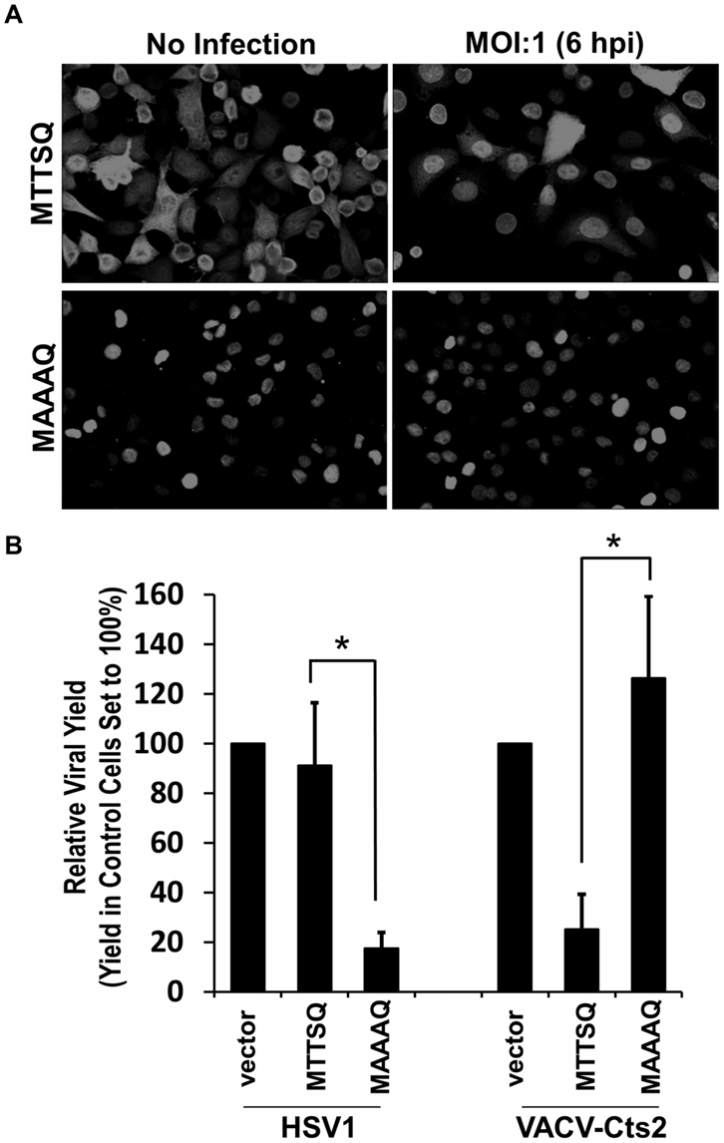
HSV-1 infection triggers relocalization of BAF to the nucleus where unphosphorylatable BAF can suppress HSV-1 viral yield. (A) Immunofluorescence analyses of FLAG-BAF-MTTSQ or MAAAQ cells infected with HSV-1 virus at MOI = 1 for 6 h. Following fixation, cells were then stained with an anti-FLAG antibody (AlexaFluor488-conjugated). (B) Viral yield analyses of HSV-1 and VACV Cts2-infected cells. Indicated cell lines were infected with HSV-1 or VACV Cts2 virus at MOI = 0.01 for 48 h at 37°C. Viral yield was calculated relative to vector control for each infection (n = 3). Error bars represent standard deviations (*indicates a p-value<0.05 from t-test analysis).

Having observed that the phosphorylation of BAF is altered by HSV-1, we sought to examine how this might affect BAF as well as the viral lifecycle. We posited that either cellular pathways are working to activate BAF’s antiviral DNA binding activity or perhaps that the virus is actively triggering BAF dephosphorylation to somehow enhance virus replication. First, we were interested in whether the decrease in BAF phosphorylation we observed in Figure 1 correlates with a change in its localization, as we saw with FLAG-BAF-MAAAQ in Figure 3A. For these studies, examination of FLAG-BAF-MTTSQ localization was used because of the greater sensitivity of the FLAG antibody in this assay. Indeed, by 6 hpi FLAG-BAF-MTTSQ clearly shifted to a more prominent nuclear localization (Figure 4A). In contrast, FLAG-BAF-MAAAQ is exclusively nuclear both in the presence and absence of HSV-1, thus demonstrating that its localization remains unchanged by infection. Together, these data provide the first evidence that BAF becomes both dephosphorylated and re-localized during the course of HSV-1 infection.

To test whether dephosphorylated BAF affects viral yield, we infected FLAG-BAF-MAAAQ-expressing cells as well as control and cells expressing FLAG-BAF-MTTSQ at a low MOI (0.01), followed by harvesting intracellular virus at 48 hpi, and measuring viral progeny by plaque titration assay. For comparison purposes, parallel infections were performed on the same cell types with the vaccinia Cts2 mutant virus, at the same MOI and time of harvest. The Cts2 virus expresses a defective form of the vaccinia B1 kinase, making it highly sensitive to BAF because the virus is unable to phospho-inactivate the antiviral activity of BAF [56], [72], [73]. In regard to the HSV-1 infections, we found that the amount of virus produced did not differ in cells overexpressing FLAG-BAF-MTTSQ when compared to control cells (Figure 4B). Strikingly however, in cells expressing the nuclear localized unphosphorylated FLAG-BAF-MAAAQ protein, viral yield was reduced more than 80%. As expected from our work in another cell line [65], examination of Cts2 virus production in these cells yielded the opposite results. Overexpression of the more cytoplasmic FLAG-BAF-MTTSQ decreased Cts2 viral yield ∼80%, while expression of FLAG-BAF-MAAAQ had no measurable impact on Cts2 infection. Together, these data provide evidence that BAF is clearly capable of host defense activity against vaccinia and it is dephosphorylated and relocalized in the presence of HSV-1. Furthermore, when BAF is not post-translationally modified at its N-terminus, as in the FLAG-BAF-MAAAQ mutant, it can interfere significantly with HSV-1 productive infection.

### BAF Binds HSV-1 DNA and Impairs Viral DNA Replication

**Figure 5:**
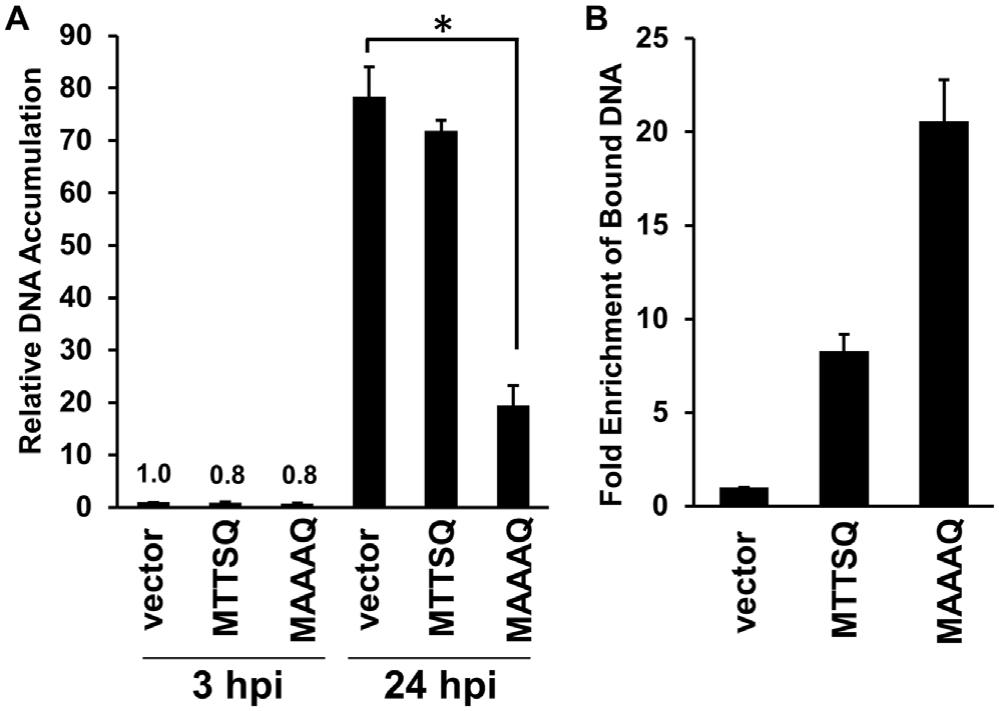
Unphosphorylatable BAF mutant suppresses HSV-1 viral DNA replication and exhibits increased relative binding to HSV-1 viral DNA. (A) Relative viral DNA accumulation of HSV-1-infected FLAG-BAF-MTTSQ and MAAAQ cells. Cells were infected with HSV-1 virus at MOI = 0.1 for 3 h and 24 h. Following harvest, cells were analyzed for viral DNA accumulation via qPCR by using HSV-1 DNA-specific primers. DNA accumulation was calculated relative to vector control at 3 hpi (n = 3). Error bars represent standard deviations. (* indicates a p-value<0.05 from t-test analysis) (B) ChIP analyses of viral DNA bound to FLAG-BAF-MTTSQ and MAAAQ HSV-infected cells. Cells were infected with HSV-1 virus at MOI = 1 for 6 h followed by fixation, immunoprecipitation with anti-FLAG antibody, and reverse crosslinking of protein-DNA complexes. Purified DNA was analyzed by qPCR using primers specific for the ICP0 promoter region. Fold enrichment was obtained relative to empty vector control and normalized to input DNA. Data were obtained from three independent experiments performed in triplicate wells and data from a representative experiment is shown. Error bars represent standard deviations.

During infection with the Cts2 vaccinia virus, BAF binds to viral DNA and interferes with viral DNA replication [56]. To test the hypothesis that FLAG-BAF-MAAAQ may be acting through a similar mechanism against HSV-1, we monitored viral DNA accumulation in control cells and cells overexpressing FLAG-BAF-MTTSQ and FLAG-BAF-MAAAQ (Figure 5A). Using real time qPCR analysis, we determined that similar amounts of viral DNA were present in all three cell types at 3 hpi. By 24 hpi, HSV-1 DNA levels in control cells had increased 80-fold, and had increased over 70-fold in cells expressing FLAG-BAF-MTTSQ. However, viral DNA accumulation was inhibited greater than 75% in cells expressing FLAG-BAF-MAAAQ as compared to controls. Next, we examined whether FLAG-BAF-MTTSQ or FLAG-BAF-MAAAQ bind viral DNA during HSV-1 infection as they do during vaccinia infection. Indeed, following FLAG-specific chromatin immunoprecipitation we found that overexpression of FLAG-BAF-MTTSQ led to an 8-fold enrichment of the ICP0 promoter as compared to control cells when measured by qPCR (Figure 5B). Enrichment of viral DNA following immunoprecipitation of FLAG-BAF-MAAAQ was 20-fold, even greater that that observed in FLAG-BAF-MTTSQ expressing cells, despite the fact that FLAG-BAF-MAAAQ was expressed at lower levels (shown earlier in Figure 1). Collectively, these data support our hypothesis that BAF-MAAAQ impairs viral production, at least in part, by binding to the viral genome more efficiently than BAF-MTTSQ, thus leading to an impairment of genome replication.

### BAF Impairs Expression of a Subset of Viral Proteins, Including ICP0

**Figure 6:**
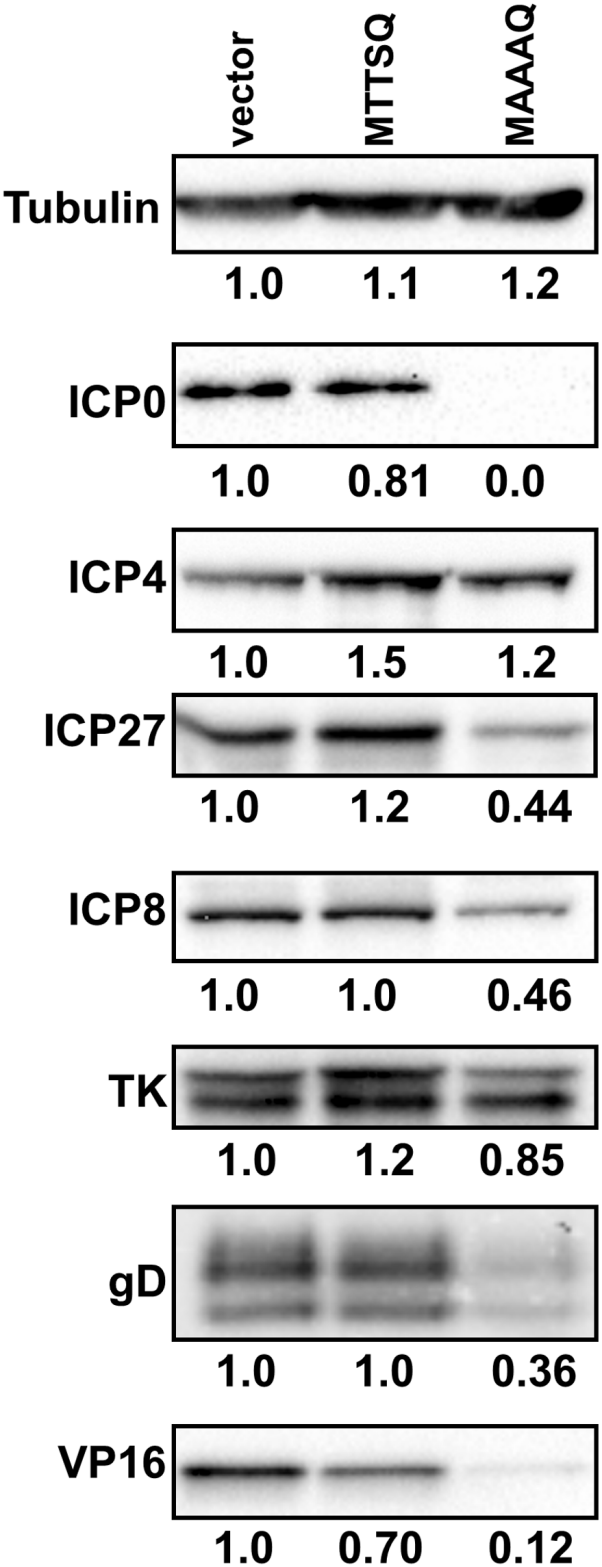
Decreased HSV-1 viral protein expression can be observed in unphosphorylatable BAF mutant cells. Western blot analysis of HSV-1 viral proteins upon infection of FLAG-BAF-MTTSQ and FLAG-BAF-MAAAQ cells. Cells were infected with HSV-1 virus at MOI = 1 for 20 h. Following cell harvest, protein samples were resolved by SDS-PAGE and transfer to membrane. Membranes were incubated with antibodies against HSV-1 proteins: ICP0, ICP4, ICP27, ICP8, TK, gD, and VP16. Anti-tubulin antibody was used as a loading control. Data were obtained from three independent experiments and a representative blot is shown. Values represent average relative signal intensity from three experiments as quantified by ImageLab software (BioRad).

In addition to inhibiting poxviral DNA replication, BAF is also capable of repressing transcription from viral promoters during infection with the Cts2 vaccinia virus [57]. To determine whether HSV-1 gene expression may also be affected by FLAG-BAF-MTTSQ or FLAG-BAF-MAAAQ, we performed western blot analysis of viral proteins following infection of control cells or cells expressing FLAG-BAF-MTTSQ or FLAG-BAF-MAAAQ. For these studies, the designated L929 cells were infected with HSV-1 (MOI = 0.1) in three independent experiments. At 20 hours after infection, the levels of viral proteins were examined by western blot analysis and average differences in protein expression calculated from those three assays. Seven viral proteins were selected as representative examples of IE (ICP0, ICP4, ICP27), E (ICP8, HSV-TK), and L (gD, VP16) genes (Figure 6). Following infection of cells expressing FLAG-BAF-MAAAQ, there were substantial changes in protein levels, with clear decreases in certain members of all three kinetic classes. Most strikingly, the levels of the E3 ubiquitin ligase/co-activator protein ICP0 were essentially undetectable at 20 hours after infection. Furthermore, the co-activator VP16, was present in FLAG-BAF-MAAAQ-expressing cells at an average of only 12% (+/−8% s.d.) of the level found in control cells. Interestingly, there appeared to be differential impacts of FLAG-BAF-MAAAQ on expression of specific genes. For example, in contrast to ICP0 or VP16, expression of HSV-TK and the transcriptional regulator ICP4 were largely unaffected, while the ICP27, ICP8, and gD proteins were all reduced 55–65%. By comparison, total cell lysate from FLAG-BAF-MTTSQ-expressing cells contained equal or greater amounts of ICP4, ICP27, ICP8, HSV-TK and gD, but contained only 70–80% of the ICP0 and VP16 found in control cells. In sum, these data argue that unphosphorylated BAF can inhibit the lifecycle of HSV-1 in the nucleus by impairing both DNA replication and expression of viral genes essential for productive infection.

## Discussion

The DNA binding protein BAF has functions both in the nucleus and the cytoplasm of the cell. In the nucleus, BAF regulates nuclear reassembly in the late stages of mitosis [52]–[55]. Evidence can also be found for the involvement of BAF in transcriptional regulation, transposon stabilization [74], and the DNA damage response in the nucleus [47], [49], [75], [76]. In the cytoplasm, BAF can bind to foreign DNA and associates with DNA of retroviral preintegration complexes, thereby protecting it from undergoing suicidal autointegration in vitro [45], [77], [78]. The ability of BAF to bind DNA in the cytoplasm also allows it to act as a host defense against vaccinia infection. When BAF is hypo-phosphorylated, it can localize to poxviral DNA [56] and impede its replication in a manner dependent on the ability of BAF to crossbridge DNA [58]. BAF also represses transcription from vaccinia intermediate promoters; however, the ability of BAF to impair poxviral DNA replication and transcription are blocked through the action of the viral B1 kinase [56], [57]. B1 is capable of phosphorylating BAF, which inactivates the DNA binding activity and antipoxviral capability of BAF. Despite its antiviral activity in the cytoplasm, whether BAF is also capable of this function during infection by a nuclear DNA virus has been unclear.

In addressing this question, we began with the premise that the phosphorylation state of BAF could be affected by HSV-1 infection and may determine its antiviral activity in the nucleus, as phosphorylation of BAF does in the cytoplasm against vaccinia virus. Indeed, we observed that during HSV-1 infection, BAF phosphorylation is sharply reduced. This decrease in phosphorylation begins at 6 hpi and continues until almost no phosphorylated BAF can be detected. Concomitant with dephosphorylation, we found BAF to relocalize to the nucleus, indicating that BAF phosphorylation and localization are modulated during HSV-1 infection as they are during vaccinia infection. To test how BAF dephosphorylation might affect the viral lifecycle, we studied not only epitope-tagged BAF-MTTSQ, but also an altered form of the protein representing constitutively unphosphorylated BAF. Analysis of the localization and DNA binding capabilities of these proteins revealed that in uninfected cells the unphosphorylated MAAAQ protein could bind DNA as efficiently as FLAG-BAF-MTTSQ, but adopted a more nuclear localization. Similar results using these mutants have also been obtained in primate cells [65], demonstrating that phosphorylation regulates the localization of BAF and its association with DNA across species.

Next, using cell lines expressing wild-type and unphosphorylated BAF, we performed HSV-1 infections and assayed the production of new virus. We found that viral yield was consistently reduced 80% in cells expressing FLAG-BAF-MAAAQ, providing the first evidence suggesting that BAF can act as an inhibitor of HSV-1 infection. In contrast, overexpression of FLAG-BAF-MTTSQ had only a minor impact on viral yield (Figure 4B). To verify that our FLAG-BAF-MTTSQ clone is capable of antiviral activity, we also compared viral yields from the same three cell lines following infection with the vaccinia Cts2 virus. This mutant virus was employed because it lacks expression of an active form of the B1 kinase [72], [73], which we previously demonstrated was required to inactivate BAF via direct phosphorylation. Measurement of Cts2 viral progeny from these cell lines demonstrated an 80% reduction in yields in cells expressing FLAG-BAF-MTTSQ, but not cells expressing FLAG-BAF-MAAAQ. This result demonstrated that the MAAAQ mutant, which was localized primarily to the nucleus, was unable to localize to the cytoplasm even though vaccinia replication and transcription was occurring in the cytoplasm.

We found the reciprocal abilities of FLAG-BAF-MTTSQ and MAAAQ to impair HSV-1 versus Cts2 vaccinia virus intriguing for the following reasons. First, the fact that FLAG-BAF-MAAAQ can bind DNA, but does not inhibit vaccinia suggests that phospho-regulation of the localization of BAF is crucial for its anti-viral activity against that virus. Second, in regard to HSV-1, it is possible that the increased presence of FLAG-BAF-MAAAQ in the nucleus may enhance its antiviral activity against nuclear viruses, in general. An additional possibility is that since FLAG-BAF-MTTSQ can be phosphorylated at its N-terminus, it may have reduced activity against HSV-1 because it is modified by a cellular and/or viral kinase. If this kinase activity is ongoing during the course of infection it would antagonize dephosphorylation of BAF, thus delaying the antiviral activity of BAF past a point in the viral lifecycle when it is most effective. One example of a cellular nuclear kinase specific for BAF is known; the cellular Vaccinia Related Kinase 1 has significant homology to the vaccinia B1 kinase [79] and plays an important role in phosphorylating BAF during mitosis [63], [69]. Further studies are needed to determine whether VRK1 or other cellular/viral kinases are regulators of the antiviral activity of native BAF or FLAG-BAF-MTTSQ in the nucleus.

Upon discovering that FLAG-BAF-MAAAQ expression correlates with reduced HSV-1 yield, we hypothesized that the mechanism of action might be similar to the antipoxviral activity of BAF. Specifically, we have previously published evidence that BAF can bind to vaccinia DNA and impair both DNA replication and transcription from viral promoters [57], [58]. The data presented herein are fully consistent with this hypothesis. Specifically, BAF does bind to HSV-1 DNA as measured by ChIP assay, and the reduced accumulation of replicated viral DNA in MAAAQ-expressing cells suggests that HSV-1 DNA replication was impaired. The finding that expression of the γ2 L viral protein VP16 was inhibited in infected cells expressing unphosphorylatable BAF is consistent with reduced viral DNA replication because it is well established that γ2 L gene expression occurs after viral DNA replication [80]. Conversely, inhibition of gD protein expression in L929 cells expressing the FLAG-BAF-MAAAQ mutant is not merely the result of reducing viral DNA replication because HSV-1 gD is a prototype γ1 gene that is expressed early during infection and its expression is not dramatically altered by a viral inhibitor of DNA replication, phosphonoacetic acid for example [80]. Furthermore, reduction of viral DNA replication would not explain the finding that expression of the immediate early protein ICP0 was not observed under the conditions of these studies in L929 cells expressing the FLAG-BAF-MAAAQ mutant. Conversely, expression of ICP4, another IE transcriptional regulator was not dramatically reduced following infection of L929 cells that express the FLAG-BAF-MAAAQ mutant adding credence to our conclusion that the FLAG-BAF-MAAAQ mutant did not have the same result on expression of all viral proteins. Relative to other HSV-1 promoters (IE, E, or L), the full-length ICP0 promoter is long and contains binding sites for numerous cellular transcription factors [81]–[83] suggesting BAF may preferentially interact with one or more of these cellular factors to inactivate the ICP0 promoter. Since the ICP0 promoter also contains 10 VP16/Oct1 binding sites, a dramatic reduction in VP16, as seen in L929 cells expressing the FLAG-BAF-MAAAQ mutant, may also reduce ICP0 expression. Studies to test these predictions are in progress.

In sum, these data demonstrate for the first time that the antiviral activity of BAF is not exclusive to vaccinia in the cytoplasm, but can also be observed in the nucleus in a phosphorylation-dependent manner. Together with other recently published evidence, our studies support a model in which mitotic regulation or cellular stresses including viral infection or other insults [84] modulate BAF via phosphorylation. In that regard, it will be of interest to determine whether overlap exists between mitotic and stress signaling cascades upstream of BAF. For example, the VRK1-PP2A/PP4 signaling axis known to target BAF during mitosis [62], [85] may also be involved in the dephosphosphorylation of BAF we observed in this study. Additionally, since HSV-1 encodes two serine/threonine protein kinases (U_S_3 and U_L_13) [86]–[88], it is not unreasonable to suggest that one of these viral encoded protein kinases directly or indirectly plays a role in maintaining BAF phosphorylation to help keep it disarmed during infection.
